# Promoting Diversity in Otolaryngology Residency Programs: Underrepresented in Medicine Funding for Visiting Medical Student Electives

**DOI:** 10.1002/oto2.70109

**Published:** 2025-04-14

**Authors:** Audrey M. Abend, Shaila T. Man, Li‐Xing Man

**Affiliations:** ^1^ Rutgers Robert Wood Johnson Medical School New Brunswick New Jersey USA; ^2^ Department of Otolaryngology Head and Neck Surgery University of Rochester Medical Center Rochester New York USA; ^3^ Princeton University Princeton New Jersey USA

**Keywords:** away rotation, diversity in medicine, underrepresented in medicine, visiting medical student elective

## Abstract

**Objective:**

This study aims to report the prevalence and characteristics of underrepresented in medicine (URiM) funding for visiting medical student clerkships in Otolaryngology–Head and Neck Surgery (OHNS) residency programs in the United States.

**Study Design:**

Manual online review of Accreditation Council for Graduate Medical Education (ACGME)‐accredited OHNS residency programs as of January 2024, reflective of typical medical student search methodology.

**Setting:**

An online review.

**Methods:**

For each program, at least 2 authors captured presence of funding, the funding amount, funding origin, and eligibility criteria. Presence and amount funding were analyzed for possible associations with program type (by FREIDA™ program description), urbanization level, cost of living, and degree of racial and ethnic diversity in the program's geographic location.

**Results:**

Of 131 programs, 49 (37.4%) offered URiM funding, primarily through diversity, equity, and inclusion (DEI) entities (67.3%) or OHNS departments (32.7%). Mean funding per 1‐month rotation was $1908. Eligibility criteria varied, with 63.2% using a non‐specific URiM definition and 18.4% following the Association of American Medical Colleges definition. Funding presence did not differ by geographic region (*P* = .06), program type, urbanization level, or cost of living. However, funding amounts varied significantly by region (*P* < .01) and were significantly different between programs in diversity index 35.0% to 44.9% versus 45.0% to 54.9% and 55.0% to 64.9% (*P* = .007 and *P* = .002, respectively).

**Conclusion:**

URiM funding is available in a minority of OHNS programs, with substantial variability in funding amount and eligibility criteria. Standardized guidance on defining URiM eligibility may benefit students and institutions. Funding may correlate with local racial and ethnic diversity, warranting further research.

The US patient population is becoming increasingly diverse: a majority of Americans will belong to a group other than non‐Hispanic white by 2044.^1^ However, the health care workforce has not reflected the increased diversity of our nation's population.^2^ Substantial evidence indicates that greater diversity in health care improves clinical decision‐making, access to health care, patient satisfaction, health outcomes, and institutional financial performance.^1^, ^2^, ^3^, ^4^, ^5^ Although there has been an increase in underrepresented populations in the health care workforce, the changes have been outpaced by the evolving diversity of the population and the US physician workforce today does not adequately reflect the diversity of patients in served communities.^6^, ^7^

In 2009, the Liaison Committee on Medical Education directed US allopathic medical schools to demonstrate a commitment to establishing programs aimed at attracting and retaining students from diverse backgrounds, such as pipeline and academic enrichment programs.^8^ Medical schools thus have developed strategies to improve medical student diversity by establishing diversity, equity, and inclusion (DEI) departments, developing new administrative positions, and establishing health equity curricula and funding opportunities for students underrepresented in medicine (URiM). Providers who are “underrepresented in medicine” generally refers to those who are historically minorities within the medical field.^9^

Funding programs for URiM students are vital for promoting access to a greater variety of medical specialties, physician networking opportunities, and career development experiences. One of the ways specialty exposure is achieved during medical school is by participating in an away rotation, otherwise known as a visiting clerkship, sub‐internship, or acting internship. Students rotate at a different medical school, where they engage with attendings and residents and gain a firsthand view of what a potential residency at the institution would entail. Additionally, studies have shown that program directors view away rotations as a key criterion in applicant evaluation, often influencing interview invitations and acceptance decisions.^10^, ^11^, ^12^, ^13^, ^14^, ^15^, ^16^, ^17^, ^18^

Participating in an away rotation program is arguably even more essential for URiM students interested in applying to Otolaryngology–Head and Neck Surgery (OHNS) residency, as both one of the most competitive specialties and one with historically poor diversity.^19^, ^20^ Between 2010 and 2018, OHNS residency programs had the lowest percentage (8.5%) of URiM matriculants of any surgical specialty.^2^ Another study of 2018 data showed that the proportion of Hispanic/Latinx (9.4%) and black medical student applicants (6.1%) to OHNS residency programs was higher than those in OHNS residencies (6.2% and 2.3%, respectively), whereas the proportion of white OHNS residents (66.2%) increased compared to OHNS applicants (51.3%).^21^ This is augmented by research that show, from 1975 to 2010, African American representation in OHNS decreased by 2.3% annually, Native American representation remained low with a growth rate of only 1.5% per year, and Hispanic representation increased at 17.3% annually, half the rate of growth observed in the general population.^22^

It is clear that OHNS is in need of URiM applicants to improve diversity, but the question is how to best improve URiM interest and participation with OHNS in medical school. Most OHNS residency applicants apply with at least 1 away rotation and URiM students considering application to OHNS residency have been shown to value mentorship, early exposure, and personal interaction with OHNS faculty, all of which are aspects of the away rotation.^15^, ^16^, ^23^, ^24^ However, these rotations can prove to be logistically challenging for many students. Financial limitations, difficulty securing housing or transportation, and potentially increased living costs in a different city may dissuade a student from pursuing an away rotation.^2^, ^11^, ^24^

## Objectives

Our primary objective was to compile and assess URiM funding opportunities from US Accreditation Council for Graduate Medical Education (ACGME)‐accredited OHNS programs, as available by online search, to reflect the opportunities that could be readily found by medical students. Additionally, we aimed to better characterize the differences between institutional parameters for students to qualify for URiM funding. This included determining whether they utilized the Association of American Colleges (AAMC) URiM definition, a “self‐determined” URiM qualification, or had additional criteria that students should meet. Additionally, we described variations in residency program characteristics and community demographics of their locations. We assessed whether the program type in the American Medical Association Residency & Fellowship Database (FREIDA™) and urban–rural classification, cost of living (COL), and the racial and ethnic diversity index (DI) score associated with the county in which the residency program was located were related to presence and amount of funding.

## Methods

**Figure 1:**
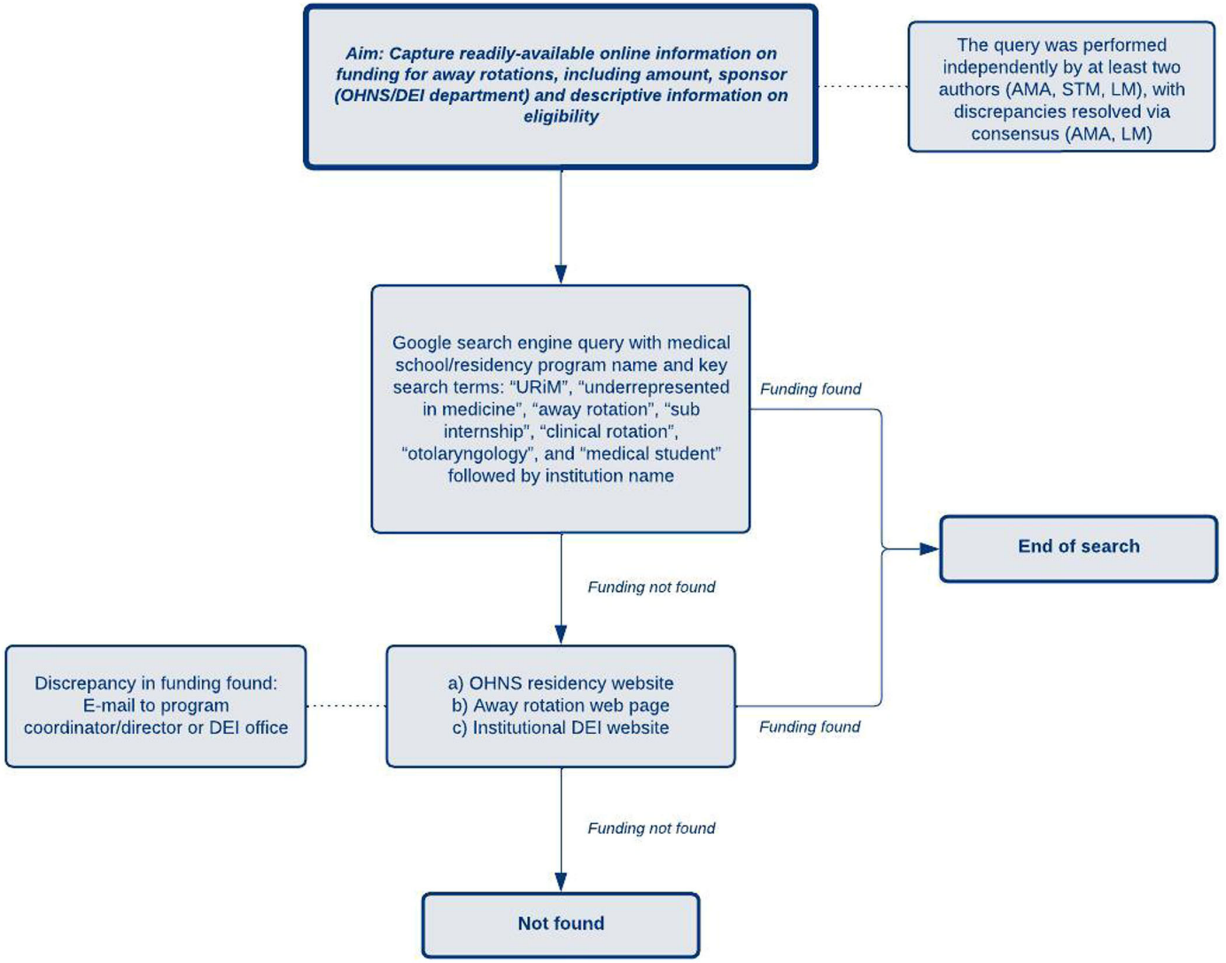
Online search methodology for URiM opportunities.

We conducted a manual, online search of every medical school with an associated and ACGME‐accredited OHNS residency program as of January 20, 2024. This project was exempt from IRB oversight as this did not involve patient data and was solely dependent on existing and public data. For each school, we captured whether there was funding for away rotations, the funding amount, whether the funding is for OHNS/any specialty rotation/both, as well as captured descriptive information on who is eligible to receive funding (Figure 1). Search methodology comprised of a primary Google search engine query conducted using variations of key search terms, including “URiM,” “underrepresented in medicine,” “away rotation,” “sub internship,” “clinical rotation,” “otolaryngology,” and “medical student” with each of the 131 ACGME‐accredited OHNS residency programs. This approach aimed to improve the efficiency of our search by often leading to an existing webpage for a URiM away rotation at an institution. If no direct link appeared on the first page of the primary Google search, we then searched the OHNS residency website, the away rotation page, and the institutional DEI website. If discrepancies in funding amounts were found, we emailed the program director, coordinator, and/or DEI office staff as needed and made reasonable efforts to follow up with the residency program for clarification. If no information could be found, the outcome was noted as “Not found,” to account for institutions non‐obvious, inquiry‐dependent, or outdated information. Of note, most schools did not publicize the number of positions available for funding. For determining the average funding amount, we used the highest amount listed for schools offering a range and the minimum listed for schools without a range. The query was performed independently by at least 2 authors (A.M.A., S.T.M., and L.M.), with discrepancies resolved via consensus (A.M.A. and L.M.). The University of Rochester's institutional review board, the Research Subjects Review Board (RSRB), within its Office for Human Subject Protection (OHSP) maintains oversight over all human subject research conducted by University of Rochester employees or agents. As this research is not human subject research, it does not fall within the scope of the RSRB per OHSP Policy 301.

Geographical regions were categorized by the AAMC‐defined regions in the Geographical Preferences section of the MyERAS application: New England (Connecticut, Maine, Massachusetts, New Hampshire, Rhode Island, and Vermont), Middle Atlantic (New Jersey, New York, and Pennsylvania), East North Central (Illinois, Indiana, Michigan, Ohio, and Wisconsin), West North Central (Iowa, Kansas, Minnesota, Missouri, Nebraska, North Dakota, and South Dakota), South Atlantic (Delaware, District of Columbia, Florida, Georgia, Maryland, North Carolina, Puerto Rico, South Carolina, Virginia, and West Virginia), East South Central (Alabama, Kentucky, Mississippi, and Tennessee), West South Central (Arkansas, Louisiana, Oklahoma, and Texas), Mountain (Arizona, Colorado, Idaho, Montana, Nevada, New Mexico, Utah, and Wyoming), and Pacific (Alaska, California, Hawaii, Oregon, and Washington). Categorical variables are reported as frequencies and percentages and continuous variables are reported as mean with standard deviation for normally distributed variables. Normality was tested with the Shapiro–Wilk test. Chi‐square test of independence was used to test the association between categorical variables and binary variables. Analysis of variance (ANOVA) used to compare normally distributed continuous variables in greater than 2 groups. A *P* value less than .05 was considered statistically significant. R (R Core Team, Vienna, Austria) and Microsoft Excel (Microsoft Corporation, Redmond, WA) were used for all analyses.

For program type, we used the program self‐description in the FREIDA™ residency program database, consisting of “Program best described as” the 5 categories: “Community‐based,” “University‐based,” “Community‐based university affiliated,” “Military‐based,” and “Other.”^25^ To assess the degree of urbanization of a residency program's location, we utilized Rural–Urban Commuting Area (RUCA) codes, which are a measure of urbanization, population density, and journey‐to‐work commuting. RUCA codes were developed jointly by the Federal Office of Rural Health Policy (FORHP), US Department of Agriculture's Economic Research Service (ERS), and WWAMI Rural Health Research Center (WWAMI RHRC) and are frequently used in health services research.^26^, ^27^ The COL for a 1‐month visiting student clerkship was approximated using the cost for a family of 1 person to live in the county in which the residency program was located via the Economic Policy Institute Family Budget Estimator.^28^ COL was categorized into quintiles to improve interpretability. Racial and ethnic diversity of the county in which the residency program was located was determined using the 2020 US Census Bureau data for the DI. The DI is the probability that 2 people chosen at random will be from different race and ethnicity groups, where a value of 0% indicates population racial and ethnic homogeneity and a value of 100% indicates that the entire population has different racial and ethnic characteristics.^29^ The DI was categorized in accordance to US Census Bureau's groupings: less than 35.0%, 35.0% to 44.9%, 45.0% to 54.9%, 55.0% to 64.9%, and 65% or more.

Residency program funding was analyzed in relation to FREIDA™ classifications, COL, and DI categories. Funding was assessed both in terms of presence or absence and as a continuous variable for programs with disclosed funding amounts (n = 48). Chi‐square tests were used to assess associations between categorical variables when expected cell counts were sufficiently large (all expected values ≥5). When expected cell counts were small (any expected value <5), Fisher's exact test was used. Categorical variables with potential non‐linear trends were analyzed using one‐way ANOVA, and if significant, Tukey's Honest Significant Difference (HSD) post hoc test was applied to identify pairwise differences between DI categories. Continuous variables were assessed with linear regression analysis.

## Results

As of January 2024, there were 131 total ACGME‐accredited OHNS residency programs (Supplemental Table 1, available online). Of those, 49 (37.4%) programs have away rotation funding for URiM students advertised online. Away rotation funding was sponsored by either an institutional DEI entity (33 programs, 67.3%) or the OHNS department itself (16 programs, 32.7%). The range of funding found was $500 to $4000 and the mean and mode amount of funding was $1908 and $2000, respectively, per 1‐month rotation. Most programs did not stipulate the number of funding opportunities offered. Two (4.1%) programs offer housing in addition to monetary funding. Seven programs (14.3%) offered additional support in the form of professional development, cultural affinity groups, or mentorship for visiting students. No programs listed the maximum number of URiM funding awards per cycle. Nine (6.9%) programs were contacted via e‐mail to resolve funding discrepancies or incomplete information found online. In terms of student eligibility for funding, 31 (63.3%) programs utilized a broad, non‐specific definition of URiM, 25 (51.0%) specified additional criteria (eg, LGBTQIA+, women, those demonstrating a commitment to diversity), 9 (18.4%) specified the AAMC definition of URiM, and 7 (14.3%) delineated students from specific racial and ethnic groups.

The New England and Pacific regions had the highest proportions of programs with URiM funding and highest mean funding was also highest in those states. No programs in the West South Central region had funding. The lowest amount of funding was in the East South Central and South Atlantic programs (Table 1). Chi‐squared analysis demonstrated no statistically significant association between AAMC‐defined geographical region and presence of funding (*P* = .06). ANOVA analysis demonstrated a correlation between region and mean amount of funding (*P* < .01). Utilizing pairwise comparison of means, the Pacific region had significantly higher funding than the South Atlantic region (*P* = .0162) and the East North Central region had significantly higher funding than the South Atlantic region (*P* = .0469). Ninety‐four OHNS residency programs were classified as “University‐based,” 22 as “Community‐based university affiliated,” 7 as “Community‐based,” 6 as “Military‐based,” and 2 as “Other” by FREIDA™. There was no significant association between FREIDA™ designation and the presence of funding (*χ*²(4) = 1.62, *P* = .80) nor did the amount of funding did not significantly differ across FREIDA™ classifications among funded programs (*F*(3, 44) = 1.59, *P* = .206).

**Table 1:** Summary of Program URiM Away Rotation Funding by AAMC‐Defined US Geographic Regions

Region	Number of programs	Number of programs with funding	Percentage of programs per region with funding	Mean funding	Mean funding (SD)
East North Central	25	9	36.0%	$2111.11	750
East South Central	6	2	33.3%	$1250.00	354
Middle Atlantic	24	8	33.3%	$1875.00	443
Mountain	6	3	50.0%	$1666.67	764
New England	9	6	66.7%	$2166.67	516
Pacific	15	8	53.3%	$2250.00	267
South Atlantic	22	8	36.4%	$1250.00	598
West North Central	9	4	44.4%	$2200.00	263
West South Central	15	0	0.0%	NA	NA

In terms of urbanization, 129 (98.5%) of otolaryngology residency programs are in the most urban Metropolitan area cores (RUCA code 1), with the remaining 2 programs in Micropolitan cores (RUCA code 4), typically a town or small city with a population between 10,000 and 49,999.^26^, ^27^ Since the overwhelming majority of programs were in Metropolitan area cores, further analysis as to the funding differences per urbanization type was not warranted.

County‐level cost of living estimates were available for all OHNS programs except for the University of Puerto Rico. The COL estimates varied across programs, with an average monthly COL of $4305.67 and an interquartile range (IQR) of $977.25 (spanning from $3669.75 to $4647). There was no significant association between 1‐month COL quintiles and presence of funding (Fisher's exact test, *P* = .313), nor were COL quintiles strong predictors of funding amount among funded programs, as the overall linear regression model was not statistically significant (*F*(4, 43) = 1.40, *P* = .249, *R*² = .115). However, when examining individual quintiles, the highest COL quintile (Q5) was significantly associated with greater funding (*β* = 467.80, SE = 204.92, *P* = .027), whereas funding amounts did not differ significantly in other quintiles (all *P* > .05).

Examining the DI, OHNS residency programs located in counties with DI 35.0% to 44.9% provided the highest average funding ($2700.00), followed by programs located in counties with DI 65% or more ($2050.00) and those in counties with DI less than 35.0% ($2000.00). In contrast, programs in counties with DI 45.0% to 54.9% had the lowest average funding ($1516.67), with a similar amount observed for programs in counties with DI 55.0% to 64.9% ($1571.43). The largest proportion of programs fell within the 65.0% and more DI category (n = 47), while the fewest programs were in the less than 35.0% category (n = 8) (Table 2).

**Table 2:** Diversity Index Ranges and Average Funding Amounts of US Otolaryngology–Head and Neck Surgery Residency Programs Offering URiM Away Rotation Funding

Diversity index range	Number of programs (percentage with funding)	Average funding per diversity index range (USD)
<35.0%	8 (37.5)	$2000.00
35.0%‐44.9%	13 (38.5)	$2700.00
45.0%‐54.9%	21 (28.6)	$1516.67
55.0%‐64.9%	42 (35.7)	$1571.43
65.0%+	47 (40.4)	$2050.00

The association between DI category and funding presence was not statistically significant (Fisher's exact test, *P* = .166), suggesting that the likelihood of receiving funding did not significantly differ by DI category. However, further analysis of DI categories revealed a significant difference in funding amounts groups amongst funded programs (*F*(4, 43) = 4.40, *P* = .0045). Post hoc Tukey's HSD test indicated that programs in the 35.0% to 44.9% DI category received significantly higher funding than those in the 45.0% to 54.9% category (mean difference = –1183.33, *P* = .0091) and the 55.0% to 64.9% category (mean difference = –1066.67, *P* = .0052). No other pairwise comparisons were statistically significant (all *P* > .05).

## Discussion

Recent years have seen a stronger focus on recruiting and retaining URiM students in the OHNS pipeline to diversify the workforce. A 2024 survey administered to all ACGME‐accredited OHNS program leadership indicated 59.5% of a total 42 respondents felt that there was a significant emphasis on URiM recruitment at their home institution. Interestingly, however, the authors found a discrepancy between perceived effectiveness of recruitment strategies and reported increase in URiM retention. Furthermore, of the strategies surveyed, none examined the effectiveness—perceived or actual—of more “upstream” efforts in the pre‐residency pipeline.^30^ To more effectively address lack of diversity in OHNS, residency leadership must address barriers earlier in the pre‐residency pipeline, such as limited access to away rotations, and incorporate these into DEI initiatives. Thompson‐Harvey et al (2022) provided key insights into URiM perceptions of OHNS as a specialty through the largest survey to date of URiM students, identifying factors that encourage or deter applicants. Their findings echoed those of previous surveys: URiM students highly value race‐concordant mentorship, research involvement, and strong relationships within their OHNS departments, with personality fit as a significant consideration, results supported by other surveys of URiM students.^20^, ^31^

Our research examined away rotations as a critical factor for prospective OHNS applicants, revealing limited access to funding, which may ultimately impact mentorship, research involvement, and opportunities to assess personality fit in the specialty. In our search of 131 ACGME‐accredited OHNS residency programs, we found a limited number of institutions offering financial support, with only 49 programs identified with funding opportunities. Aside from no identified URiM funding opportunities in the West South Central region, we did not find any regional differences in availability of funding opportunities. However, there was a significant difference in mean amount of funding by region. Specifically, higher funding in the Pacific and East North Central regions as compared to the South Atlantic region. Although future analyses on a comprehensive survey of URiM funding would be necessary to determine potential contributing factors such as denser city populations, more urban areas with higher concentration of higher education and thus DEI initiatives, the scarcity may contribute to lower URiM participation in these rotations, and ultimately URiM representation in OHNS. It is important to note that national organizations have implemented targeted efforts to support URiM students through away rotation scholarships, which may augment institutional‐level weaknesses. The American Academy of Otolaryngology–Head and Neck Surgery (AAO‐HNS) Diversity Endowment URM Away Rotation Grant offers $1000 to 2 URiM students to help offset the costs of participating in away rotations.^32^ Furthermore, the AAO‐HNS provides up to 18 additional opportunities per year sponsored by industry partners. Similarly, the Society of University Otolaryngologists (SUO) URiM Away Rotation Scholarship provides funding in the amount of $2000 to promote URiM student access to critical experiences OHNS programs.^33^ Such national grants are particularly valuable given that only 49 of 131 OHNS residency programs were found to advertise their URiM‐specific funding online.

Additionally, we found each institution had their own qualifications for funding. These ranged from the required rotations students must have completed in home clinical programs, to citizenship status, and how the school defined URiM students. Before 2004, the AAMC specifically delineated “Blacks, Mexican‐Americans, Native Americans (that is, American Indians, Alaska Natives, and Native Hawaiians), and mainland Puerto Ricans” as students who are considered URiM. In 2004, the AAMC expanded the definition of URiM to include “those racial and ethnic populations that are underrepresented in the medical profession relative to their numbers in the general population.”^34^ While this definition is more inclusive, it does not include all minorities or socioeconomically disadvantaged groups, especially as the population has continued to diversify in the last 20 years. Nevertheless, our findings that URiM funding programs have not been consistent with the use of either AAMC URiM definition. The definition of URiM varied widely, and included the AAMC URiM definition, a broader definition that included students who self‐identified as URiM, women, people in the LGBTQIA+ community, people with disabilities, students committed to diversity, and other specific ethnic and racial groups as members of URiM. Specifically, of 49 total programs found offering URiM funding for OHNS, only 9 programs utilized the updated AAMC definition as inclusion criteria for student acceptance. The variance in the standards under which medical students qualify for away rotation funding underscores the need to reassess what it means to be URiM in America. Ultimately, the differences between medical schools' definitions may be confusing for students who would qualify as URiM at one school but not another, and dissuade them from applying to additional away rotations. In changing their definition, the AAMC cited the desire to shift the definition to match the dynamic nature of the nation's diverse population—as such, almost 20 years later, it is likely that there are more ways to define URiM as a subset of the medical professional population.

Moreover, beyond the practical considerations involved in adopting a more uniformly inclusive definition for URiM, there is a need to address potential legal implications associated of not doing so. In June 2023, the Supreme Court of the United States (SCOTUS) ruled that affirmative action in higher education was unconstitutional.^35^ In response to the court's ruling, higher education institutions were required to cease directly considering race as a positive factor for applicants from historically underrepresented groups. Additionally, over the past 5 years, various medical schools have faced legal actions accusing them of engaging in illegal race‐based or sex‐based discrimination in URiM scholarship or clerkship programs across different medical specialties.^36^, ^37^, ^38^, ^39^ In response, the institutions revised or removed these programs from their official platforms. In response to the SCOTUS ruling, medical schools are likely increasingly proactive with regard to modifying the language associated with advertised URiM scholarship programs to be more inclusive of all underrepresented populations while still pursuing DEI goals. Furthermore, the SCOTUS rulings on the use of affirmative action in undergraduate admissions have already affected the holistic review process for medical school admissions as well as residency and fellowship matches.^40^, ^41^

To contextualize our findings, we also sought to assess the relationship between both program‐level (FREIDA™ classification) and location‐level (urbanization, COL, and DI) characteristics with the presence and amount of funding. As expected, the overwhelming majority of programs were located in the most urbanized environments. Additionally, neither FREIDA™ classification nor COL quintiles demonstrated a meaningful relationship with funding presence or amount, although funding was consistently below the estimated COL for a 1‐month rotation, with the exception of a singular program. However, DI categories were significantly associated with funding amounts, and notably, programs located in counties with DI 35.0% to 44.9% had significantly higher funding compared to those in counties with DI in the 45.0% to 54.9% and 55.0% to 64.9% ranges, highlighting a potential disparity in funding amount among programs located in areas of varying racial and ethnic diversity. These results underscore the need for further investigation into how locoregional racial and ethnic diversity and subsequent institutional diversity metrics may influence financial support for URiM trainees.

There were limitations in our search. Search methodology was limited to using permutations of what the authors deemed as the most appropriate search terms to elicit the URiM funding page and/or residency page. Of note, the goal of our search methodology was to reflect that of a typical medical student searching for information online and not to exhaustively verify all available funding individually with programs. Thus, it was not possible to definitively note the lack of funding at a school if it could not be found using our search methodology. Additionally, our search focused on Otolaryngology–Head and Neck residency funding. Future directions would include a more comprehensive search by administering a survey of all ACGME‐accredited OHNS residency programs to assess presence and amount of URiM funding, the reason for lack of funding or any initiatives that may be in progress, and funding guidelines to establish a more comprehensive database for medical students. The AAMC currently hosts a database within their Visiting Student Learning Opportunities™ (VSLO®) program, which purports to capture all available URiM funding opportunities for medical students in an easily accessible, centralized location.^42^ However, a preliminary manual search of this database highlights the fact that the database is neither comprehensive nor user‐friendly. The “Specialty” drop‐down selection does not include “Otolaryngology” as an option, nor do all OHNS scholarship opportunities show up for an individual program that is selected. Additionally, metrics such as the amount of funding provided, qualification for funding, and definition of URiM were not readily available. Our additional research may be used to augment the existing VSLO® URiM funding database.

## Conclusion

There is significant evidence that diversity in health care is of inherent value to workplace culture, patient care, and institutional success. So much so that, in 2019, the ACGME mandated that residency and fellowship programs improve the diversity of their workforce. However, programs were provided few guidelines for improving recruitment of URiM applicants, resulting in heterogeneous efforts of unclear value. Thus, medical schools have struggled to match the level of multifactorial diversity that exists within the patient population with medical doctors. One area of improvement that medical schools have focused on is funding for programs that provide exposure to different specialties and residency programs for URiM students, such as the away rotation. As one of the most competitive specialties to match into and one of least diverse by the numbers, insight into the prevalence of such funding for OHNS residency programs provides insight into potential areas of improvement for medical schools for increasing URiM residents in the specialties, which are most lacking. URiM funding availability has the capacity to affect student's willingness and ability to explore a specific rotation. Lack of funding may also help to explain why OHNS program and other specialty programs have lower numbers of URiM‐identifying students matriculating. In our search, we found a relative lack of funding opportunities for URiM students for otolaryngology residency programs when compared to other residency programs. Interestingly, we also found significant variation in the level of funding between institutions, as well as how students qualify for funding and who qualifies as a URiM student. To improve URiM funding utilization as well as inter‐school program consistency, it may be time for the AAMC to update their guidelines for what it means to be URiM.

## Author Contributions

**Audrey Abend**, conception, data collection, data analysis, manuscript drafting, manuscript edits, manuscript finalization, manuscript submission; **Shaila Man**, data collection; **Li‐Xing Man**, conception, data collection, data analysis, manuscript edits, manuscript finalization.

## Disclosures

### Competing interests

None.

### Funding source

None.

## Supporting information

Supporting Information.
