# Supplementary material for: Promoting Diversity in Otolaryngology Residency Programs: Underrepresented in Medicine Funding for Visiting Medical Student Electives
Source: OTO Open. 2025 Apr 14;9(2):e70109. doi: 10.1002/oto2.70109 (PMC11995422; doi:10.1002/oto2.70109)
Supplement: Supplementary file 1 — Supporting Information. [file OTO2-9-e70109-s001.docx]

| Residency Program | Medical School | URiM Funding OHNS Sub-I (OHNS-sponsored, DEI-sponsored, N) | Amount of funding (USD) (upper limit of range or highest amount stated) | Specifically, Black/African-American, Hispanic/Latino, Native American/Alaska Native, and Pacific Islander/Native Hawaiian. | Self-identification of URiM by AAMC definition | Broad self-identification of URiM | Other definitions (first generation-college student, LQBTQIA+, identify as female, students with disabilities, commitment to diversity, or additional ethnicities) | Definition not specified/not found | Comments |
| --- | --- | --- | --- | --- | --- | --- | --- | --- | --- |
| University of Alabama Medical Center Program | **University of Alabama** | **DEI-sponsored** | $1,000.00 |  |  | Y |  |  | <https://www.uab.edu/medicine/surgery/education/medical-students/visiting-students> |
| University of Arkansas for Medical Sciences (UAMS) College of Medicine Program | **University of Arkansas College of Medicine** | **N** |  |  |  |  |  |  | Specific non-Otolaryngology specialties have URiM visiting clerkship funding |
| Mayo Clinic College of Medicine and Science (Phoenix) Program | **Mayo Clinic Alix School of Medicine** | **DEI-sponsored** | $2,500.00 |  |  | Y | Y |  | <https://college.mayo.edu/academics/visiting-medical-student-clerkships/scholarships/> |
| University of Arizona College of Medicine-Tucson Program | **University of Arizona School of Medicine** | **DEI-sponsored** | $1,500.00 |  |  | Y |  |  | <https://diversity.medicine.arizona.edu/gme-diversity/recruitment> |
| Loma Linda University Health Education Consortium Program | **Loma Linda School of Medicine** | **N** |  |  |  |  |  |  |  |
| Cedars-Sinai Medical Center Program | **University of California Los Angeles David Geffen School of Medicine** | **DEI-sponsored** | $2,500.00 |  |  | Y | Y |  | <https://www.cedars-sinai.edu/education/medical-students/diversity-program.html> |
| University of Southern California/LAC+USC Medical Center Program | **Keck School of Medicine** | **DEI-sponsored** | $2,000.00 |  |  | Y | Y |  | <https://keck.usc.edu/diversity-and-inclusion/diversity-in-medicine-visiting-clerkship-award/> |
| University of California Los Angeles David Geffen School of Medicine/UCLA Medical Center Program | **University of California Los Angeles David Geffen School of Medicine** | **OHNS-sponsored** | $2,000.00 |  |  | Y | Y |  | <https://www.uclahealth.org/departments/head-neck-surgery/academic-programs/visiting-student-scholarship-program> |
| Kaiser Permanente Northern California Program | **Kaiser Permanente Bernard J. Tyson School of Medicine** | **N** |  |  |  |  |  |  |  |
| University of California (Irvine) Program | **University of California, Irvine, School of Medicine Program** | **N** |  |  |  |  |  |  | [https://ent.uci.edu//residents/rotation.asp](https://ent.uci.edu/residents/rotation.asp) |
| University of California Davis Health Program | **UC Davis School of Medicine** | **OHNS-sponsored** | $2,500.00 |  | Y |  |  |  | <https://health.ucdavis.edu/otolaryngology/education/residency/endowment-flyer.pdf> |
| Naval Medical Center (San Diego) Program | **None** | **N** |  |  |  |  |  |  |  |
| University of California (San Diego) Medical Center Program | **University of California San Diego School of Medicine** | **OHNS-sponsored** | $2,500.00 |  |  | Y | Y |  | <https://oto.ucsd.edu/education/visiting-clerkship-scholarship/index.html> |
| University of California (San Francisco) Program | **University of California San Francisco Medical School** | **DEI-sponsored** | $2,000.00 |  |  | Y | Y |  | <https://meded.ucsf.edu/residents-clinical-fellows/gme-resident-and-fellow-resources/diversity-gme/visiting-elective-scholarship#SELECTION> |
| Stanford Health Care-Sponsored Stanford University Program | **Stanford Medical School** | **DEI-sponsored** | $2,000.00 |  |  | Y | Y |  | <https://med.stanford.edu/clerkships/score-program.html> |
| University of Colorado Program | **University of Colorado Medical School** | **OHNS-sponsored** | $1,000.00 |  |  | Y |  |  | <https://medschool.cuanschutz.edu/otolaryngology/education/medical-students> |
| University of Connecticut Program | **University of Connecticut Medical School** | **DEI-sponsored** | $1,500.00 |  | Y |  |  |  | <https://medicine.uconn.edu/visiting-students/vesum/> |
| Yale-New Haven Medical Center Program | **Yale Medical School** | **DEI-sponsored** | $1,500.00 |  |  | Y | Y |  | [https://medicine.yale.edu/md-program/visiting-students/us/visiting_diversity_scholarship/]( https://medicine.yale.edu/md-program/visiting-students/us/visiting_diversity_scholarship/) |
| George Washington University Program | **George Washington Medical School** | **DEI-sponsored** | $1,000.00 |  |  | Y | Y |  | [ENT is division of Surgery  https://diversity.smhs.gwu.edu/programs/visiting-clerkship-program](https://diversity.smhs.gwu.edu/programs/visiting-clerkship-program) |
| MedStar Health/Georgetown University Hospital Program | **Georgetown University Medical School** | **OHNS-sponsored** | $1,500.00 |  | Y |  |  |  | <https://ent.georgetown.edu/school-of-medicine/> |
| University of Florida Program | **University of Florida Medical School** | **N** |  |  |  |  |  |  |  |
| Mayo Clinic College of Medicine and Science Program (Jacksonville) | **Mayo Clinic Alix School of Medicine** | **DEI-sponsored** | $2,500.00 |  |  | Y | Y |  | <https://college.mayo.edu/academics/visiting-medical-student-clerkships/scholarships/> |
| University of Miami/Jackson Health System Program | **University of Miami Medical School** | **N** |  |  |  |  |  |  |  |
| University of South Florida Morsani Program | **University of South Florida Medical School** | **N** |  |  |  |  |  |  |  |
| Emory University School of Medicine Program | **Emory University School of Medicine** | **N** |  |  |  |  |  |  | <https://med.emory.edu/departments/otolaryngology/research/emory_research_group_oto/ergo_minority_oto_clerkship.html> |
| Medical College of Georgia Program | **Medical College of Georgia** | **N** |  |  |  |  |  |  |  |
| Tripler Army Medical Center Program | **None** | **N** |  |  |  |  |  |  |  |
| University of Iowa Hospitals and Clinics Program | **University of Iowa Medical School** | **DEI-sponsored** | $2,500.00 |  |  | Y |  |  | <https://medicine.uiowa.edu/diversity/studentstrainees/prospective-studentstrainees/ui-underrepresented-medicine-visiting-student-elective> |
| McGaw Medical Center of Northwestern University Program | **Feinberg School of Medicine** | **DEI-sponsored** | $1,500.00 |  | Y | Y | Y |  | <https://www.mcgaw.northwestern.edu/diversity-inclusion/visiting-clerkship-stipends.html> |
| Rush University Medical Center Program | **Rush Medical School** | **N** |  |  |  |  |  |  | [RUSH program for other specialties https://www.rushu.rush.edu/rush-medical-college/visiting-medical-students](https://www.rushu.rush.edu/rush-medical-college/visiting-medical-students) |
| University of Chicago Program | **University of Chicago Medical School** | **DEI-sponsored** | $2,000.00 |  | Y |  | Y |  | <https://pritzker.uchicago.edu/academics/underrepresented-visiting-students> |
| University of Illinois College of Medicine at Chicago Program | **University of Illinois School of Medicine** | **N** |  |  |  |  |  |  |  |
| Loyola University Medical Center Program | **Loyola University School of Medicine** | **DEI-sponsored** | Not Found |  |  |  | Y |  | <https://www.loyolamedicine.org/gme/health-equity-diversity-visiting-clerkship/> |
| Southern Illinois University Program | **Southern Illinois School of Medicine** | **N** |  |  |  |  |  |  |  |
| Indiana University School of Medicine Program | **Indiana University School of Medicine** | **DEI-sponsored** | $2,000.00 | Y |  |  |  |  | <https://mddiversity.wustl.edu/wp-content/uploads/2019/05/IU-URM-Clerkship.pdf> |
| University of Kansas School of Medicine Program | **University of Kansas School of Medicine** | **N** |  |  |  |  |  |  |  |
| University of Kentucky College of Medicine Program | **University of Kentucky School of Medicine** | **N** |  |  |  |  |  |  |  |
| University of Louisville School of Medicine Program | **University of Lousiville School of Medicine** | **N** |  |  |  |  |  |  |  |
| Tulane University Program | **Tulane University School of Medicine** | **N** |  |  |  |  |  |  |  |
| Louisiana State University School of Medicine Program | **Louisiana State University School of Medicine** | **N** |  |  |  |  |  |  |  |
| Louisiana State University (Shreveport) Program | **Louisiana State University School of Medicine** | **N** |  |  |  |  |  |  |  |
| Massachusetts Eye and Ear Infirmary/Harvard Medical School Program | **Harvard Medical School** | **DEI-sponsored** | $2,500.00 |  |  | Y |  |  | <https://dicp.hms.harvard.edu/dicp-programs/medical-and-graduate/vcp> |
| Tufts Medical Center Program | **Tufts University School of Medicine** | **N** |  |  |  |  |  |  |  |
| Boston University Medical Center Program | **Boston University School of Medicine** | **DEI-sponsored** | $2,500.00 | Y |  |  |  |  | <https://www.bmc.org/medical-professionals/office-minority-physician-recruitment/visiting-medical-students> |
| Beth Israel Deaconess Medical Center/Harvard Medical School Program | **Harvard Medical School** | **DEI-sponsored** | $2,500.00 |  |  | Y |  |  | <https://dicp.hms.harvard.edu/dicp-programs/medical-and-graduate/vcp> |
| University of Maryland Program | **University of Maryland College of Medicine** | **N** |  |  |  |  |  |  |  |
| University of Massachussetts Chan Program | **University of Massachussetts Chan Medical School** | **N** |  |  |  |  |  |  | [ENT not an option for rotation  https://www.baystatehealth.org/Education-Research/Education/UMMS-Baystate-Campus/PURCH/applying/URiM-Scholarship](https://www.baystatehealth.org/Education-Research/Education/UMMS-Baystate-Campus/PURCH/applying/URiM-Scholarship) |
| Johns Hopkins University Program | **Johns Hopkins University School of Medicine** | **OHNS-sponsored** | $500.00 |  | Y |  |  |  | <https://www.hopkinsmedicine.org/otolaryngology/education/otolaryngology-clerkships-for-medical-students#elective> |
| National Capital Consortium Program/Walter Reed National Military Medical Center | **None** | **N** |  |  |  |  |  |  |  |
| University of Michigan Health System Program | **University of Michigan Medical School** | **DEI-sponsored** | $2,000.00 |  |  | Y | Y |  | <https://www.michiganmedicine.org/diversity-equity-inclusion/students-pathways/medical-school-programs/health-equity-visiting-clerkship> |
| Henry Ford Macomb Hospital Program | **Michigan State University College of Osteopathic Medicine** | **N** |  |  |  |  |  |  |  |
| Detroit Medical Center Corporation Program | **Wayne State University Medical School** | **N** |  |  |  |  |  |  |  |
| Henry Ford Health/Henry Ford Hospital Program | **Wayne State University Medical School** | **N** |  |  |  |  |  |  |  |
| Wayne State University School of Medicine Program | **Wayne State University Medical School** | **N** |  |  |  |  |  |  |  |
| Beaumont Health (Farmington Hills) Program | **Michigan State University College of Osteopathic Medicine** | **N** |  |  |  |  |  |  |  |
| McLaren Health Care/Oakland/Michigan State University Program | **Michigan State University College of Osteopathic Medicine** | **N** |  |  |  |  |  |  |  |
| Ascension Macomb-Oakland Hospital Program | **Multiple (Michigan State University College of Osteopathic Medicine and Kansas City University of Medicine and Biosciences)** | **N** |  |  |  |  |  |  |  |
| University of Minnesota Program | **University of Minnesota Program** | **OHNS-sponsored** | $2,000.00 |  | Y |  |  |  | <https://med.umn.edu/ent/medical-students> |
| Mayo Clinic College of Medicine and Science (Rochester) Program | **Mayo Clinic Alix School of Medicine** | **DEI-sponsored** | $2,500.00 |  |  | Y | Y |  | <https://college.mayo.edu/academics/visiting-medical-student-clerkships/scholarships/> |
| University of Missouri-Columbia Program | **University of Missouri** | **N** |  |  |  |  |  |  | <https://medicine.missouri.edu/offices-programs/office-of-diversity-and-inclusion/visiting-student-program> |
| Kansas City University GME Consortium (KCU-GME Consortium)/Freeman Program | **Kansas City University College of Osteopathic Medicine** | **N** |  |  |  |  |  |  |  |
| St Louis University School of Medicine Program | **St Louis University School of Medicine** | **N** |  |  |  |  |  |  | [Summer Research Program (URiM) https://www.slu.edu/medicine/diversity/summer-undergraduate-research-program.php](https://www.slu.edu/medicine/diversity/summer-undergraduate-research-program.php) |
| Washington University/Barnes-Jewish Hospital/St. Louis Children's Hospital Consortium Program | **Washington University School of Medicine** | **OHNS-sponsored** | $2,100.00 |  |  | Y |  |  | <https://mddiversity.wustl.edu/wp-content/uploads/2022/06/ODP-VEP-Supplemental-Application-UPDATED-6.15.22.pdf> |
| University of Mississippi Medical Center Program | **University of Mississippi School of Medicine** | **N** |  |  |  |  |  |  |  |
| University of North Carolina Hospitals Program | **University of North Carolina School of Medicine** | **DEI-sponsored** | $1,000.00 |  |  | Y | Y |  | <https://www.med.unc.edu/inclusion/programs-initiatives/visiting-student-elective/> |
| Duke University Hospital Program | **Duke University School of Medicine** | **N** |  |  |  |  |  |  |  |
| Wake Forest University School of Medicine Program | **Wake Forest University School of Medicine** | **DEI-sponsored** | $1,000.00 |  |  | Y | Y |  | <https://school.wakehealth.edu/education-and-training/educational-excellence/diversity-and-inclusion/scholarships-and-grants/scholarship-clerkship-program> |
| University of Nebraska Medical Center College of Medicine Program | **University of Nebraska School of Medicine** | **N** |  |  |  |  |  |  |  |
| Dartmouth-Hitchcock/Mary Hitchcock Memorial Hospital Program | **Geisel School of Medicine at Dartmouth** | **DEI-sponsored** | $2,500.00 |  |  | Y | Y |  | <https://geiselmed.dartmouth.edu/md-program/offices-in-undergraduate-medical-education/clinical-education/dartmouth-visiting-student-program/> |
| Cooper Hospital-University Medical Center Program | **Cooper Medical School** | **N** |  |  |  |  |  |  |  |
| Rutgers Health/New Jersey Medical School Program | **NJMS** | **OHNS-sponsored** | $2,000.00 |  |  | Y | Y |  | <https://njms.rutgers.edu/education/registrar/urim.php> |
| University of New Mexico School of Medicine Program | **University of New Mexico School of Medicine** | **N** |  |  |  |  |  |  |  |
| Kirk Kerkorian School of Medicine at University of Nevada Las Vegas Program | **University of Nevada Las Vegas Kirk Kerkorian school of Medicine** | **N** |  |  |  |  |  |  |  |
| Albany Medical Center Program | **Albany Medical College** | **N** |  |  |  |  |  |  |  |
| Montefiore Medical Center/Albert Einstein College of Medicine Program | **Albert Einstein College of Medicine** | **N** |  |  |  |  |  |  |  |
| State University of New York Downstate Health Sciences University Program | **Downstate School of Medicine** | **OHNS-sponsored** | $2,000.00 |  | Y |  |  |  | <https://suo-aado.org/page/DivOpportunities> |
| University at Buffalo Program | **Jacobs School of Medicine** | **N** |  |  |  |  |  |  |  |
| Zucker School of Medicine at Hofstra/Northwell Program | **Zucker School of Medicine** | **N** |  |  |  |  |  |  |  |
| Icahn School of Medicine at Mount Sinai (New York Eye and Ear Infirmary/Mount Sinai) Program | **Icahn School of Medicine** | **N** |  |  |  |  |  |  |  |
| New York Presbyterian Hospital (Columbia and Cornell Campus) Program | **Multiple (Columbia and Cornell)** | **N** |  |  |  |  |  |  |  |
| New York University Grossman School of Medicine Program | **New York University Grossman School of Medicine** | **N** |  |  |  |  |  |  | <https://med.nyu.edu/departments-institutes/otolaryngology-head-neck-surgery/education/medical-student-education> |
| University of Rochester Program | **University of Rochester School of Medicine** | **DEI-sponsored** | $1,000.00 |  |  | Y | Y |  | <https://www.urmc.rochester.edu/ear-nose-throat/education/clerkships-for-urim-students-1.aspx> |
| Stony Brook Medicine Program | **Renaissance School of Medicine at Stony Brook University** | **N** |  |  |  |  |  |  |  |
| SUNY Upstate Medical University Program | **SUNY Upstate Medical School** | **N** |  |  |  |  |  |  |  |
| Westchester Medical Center Program | **New York Medical College** | **N** |  |  |  |  |  |  |  |
| St Elizabeth Boardman Hospital Program | **None** | **N** |  |  |  |  |  |  |  |
| University of Cincinnati Medical Center/College of Medicine Program | **University of Cincinnati School of Medicine** | **DEI-sponsored** | $1,500.00 | Y |  |  |  |  | [Otolaryngology included (e-mail correspondence) https://med.uc.edu/diversity/supporting-programs](https://med.uc.edu/diversity/supporting-programs) |
| Case Western Reserve University/University Hospitals Cleveland Medical Center Program | **Case Western Reserve University School of Medicine** | **DEI-sponsored** | $2,500.00 | Y |  |  |  |  | <https://www.uhhospitals.org/about-uh/diversity-and-inclusion/medical-education/david-satcher-clerkship> |
| Cleveland Clinic Foundation Program | **Cleveland Clinic Lerner College of Medicine** | **DEI-sponsored** | $2,000.00 |  |  | Y | Y |  | <https://my.clevelandclinic.org/departments/elective-program/scholarship-for-underrepresented-students> |
| Ohio State University Hospital Program | **Ohio State University School of Medicine** | **OHNS-sponsored** | $1,500.00 |  |  | Y |  |  | <https://medicine.osu.edu/departments/otolaryngology/education/medical-students> |
| Ohio Health/Doctors Hospital Program | **None** | **N** |  |  |  |  |  |  | <https://www.ohiohealth.com/medical-education/physician-diversity-scholars-program> |
| Western Reserve Hospital Program | **Lake Erie College of Osteopathic Medicine** | **N** |  |  |  |  |  |  |  |
| Kettering Health Network Program | **Loma Linda School of Medicine, Ohio University, Wright State University** | **N** |  |  |  |  |  |  |  |
| University of Oklahoma Health Sciences Center Program | **University of Oklahoma College of Medicine** | **N** |  |  |  |  |  |  | [OU-Tulsa has a DEI program but no ENT residency  https://www.ou.edu/tulsa/community_medicine/currrent-students/student-services/visitingstudent/URIM](https://www.ou.edu/tulsa/community_medicine/currrent-students/student-services/visitingstudent/URIM) |
| Oklahoma State University Center for Health Sciences Program | **Oklahoma State University College of Osteopathic Medicine** | **N** |  |  |  |  |  |  |  |
| Oregon Health & Science University Program | **OHSU School of Medicine** | **N** |  |  |  |  |  |  | [Programs in Surgery, IM, Peds, EM, Ophtho, Derm  https://www.ohsu.edu/school-of-medicine/md-program/visiting-students](https://www.ohsu.edu/school-of-medicine/md-program/visiting-students) |
| Lehigh Valley Health Network Program | **University of South Florida Morsani School of Medicine** | **N** |  |  |  |  |  |  |  |
| Geisinger Health System Program | **Geisinger Commonwealth School of Medicine** | **N** |  |  |  |  |  |  |  |
| St Luke's Hospital-Anderson Campus Program | **Temple/St. Luke's School of Medicine** | **N** |  |  |  |  |  |  |  |
| University of Pittsburgh Medical Center (Erie) Program | **University of Pittsburgh School of Medicine** | **DEI-sponsored** | $2,000.00 | Y |  |  |  |  | <https://www.medschooldiversity.pitt.edu/our-programs/visiting-student> |
| Penn State Milton S Hershey Medical Center Program | **Penn State Medical School** | **N** |  |  |  |  |  |  |  |
| Philadelphia College of Osteopathic Medicine Program | **Philadephia College of Osteopathic Medicine** | **N** |  |  |  |  |  |  |  |
| Temple University Hospital Program | **Temple University School of Medicine** | **N** |  |  |  |  |  |  |  |
| Sidney Kimmel Medical College at Thomas Jefferson University/Thomas Jefferson University Hospital Program | **Sidney Kimmel Medical School** | **OHNS-sponsored** | $1,500.00 |  |  | Y |  |  | <https://www.jefferson.edu/academics/colleges-schools-institutes/skmc/departments/otolaryngology/education/medical-student-education.html> |
| University of Pennsylvania Health System Program | **Perelman School of Medicine** | **DEI-sponsored** | $2,000.00 |  |  |  |  | Y | <http://www.allianceofminorityphysicians.org/penn-visiting-clerkship-program.html> |
| Allegheny Health Network Medical Education Consortium Program | **Multiple (Drexel, Temple, Lake Erie College of Osteopathic Medicine)** | **DEI-sponsored** | $2,500.00 |  |  | Y |  |  | <https://www.ahn.org/health-care-professionals/education/career-exploration/underrepresented-minority-urim-clerkship#whybecomeclerk> |
| University of Pittsburgh Medical Center (Pittsburgh) Program | **University of Pittsburgh School of Medicine** | **DEI-sponsored** | $2,000.00 | Y |  |  |  |  | <https://www.medschooldiversity.pitt.edu/our-programs/visiting-student> |
| University of Puerto Rico Program | **University of Puerto Rico** | **N** |  |  |  |  |  |  |  |
| Medical University of South Carolina Program | **University of South Carolina Medical School** | **OHNS-sponsored** | $1,000.00 |  |  | Y | Y |  | <https://medicine.musc.edu/diversity/students/visiting-students> |
| University of Tennessee Program | **University of Tennessee Medical School** | **N** |  |  |  |  |  |  |  |
| Vanderbilt University Medical Center Program | **Vanderbilt Medical School** | **OHNS-sponsored** | $1,500.00 |  | Y |  | Y |  | <https://www.vumc.org/ent/medical-students> |
| University of Texas Southwestern Medical Center Program | **University of Texas Southwestern Medical School** | **N** |  |  |  |  |  |  |  |
| University of Texas Medical Branch Hospitals Program | **John Sealy School of Medicine** | **N** |  |  |  |  |  |  |  |
| Methodist Hospital (Houston) Program | **Multiple (Cornell, A&M, University of Houston)** | **N** |  |  |  |  |  |  |  |
| University of Texas Health Science Center at Houston Program | **McGovern Medical School** | **N** |  |  |  |  |  |  |  |
| Baylor College of Medicine Program | **Baylor College of Medicine** | **N** |  |  |  |  |  |  |  |
| San Antonio Uniformed Services Health Education Consortium Program | **None** | **N** |  |  |  |  |  |  |  |
| Texas Tech University Health Sciences Center at Lubbock Program | **Texas Tech University Health Sciences Center School of Medicine** | **N** |  |  |  |  |  |  |  |
| University of Texas Health Science Center San Antonio Joe and Teresa Lozano Long School of Medicine Program | **University of Texas Health San Antonio Joe and Teresa Lozano Long School of Medicine** | **N** |  |  |  |  |  |  |  |
| Texas A&M College of Medicine-Scott and White Medical Center (Temple) Program | **Texas A&M College of Medicine** | **N** |  |  |  |  |  |  |  |
| University of Utah Health Program | **University of Utah School of Medicine** | **N** |  |  |  |  |  |  | <https://suo-aado.org/page/DivOpportunities> |
| University of Virginia Medical Center Program | **University of Virginia Medical School** | **DEI-sponsored** | $1,500.00 |  |  | Y | Y |  | <https://med.virginia.edu/md-program/wp-content/uploads/sites/462/2023/01/Application-for-UVA-4thYear-URiM-Leadership-Program.2023.pdf> |
| Eastern Virginia Medical School Program | **Eastern Virginia Medical School** | **N** |  |  |  |  |  |  |  |
| Naval Medical Center (Portsmouth) Program | **None** | **N** |  |  |  |  |  |  |  |
| Virginia Commonwealth University Health System Program | **Virginia Commonwealth University School of Medicine** | **N** |  |  |  |  |  |  |  |
| University of Vermont Medical Center Program | **Univesity of Vermont Medical School** | **N** |  |  |  |  |  |  | <https://www.med.uvm.edu/diversityinclusion/initatives/vsesp> |
| University of Washington Program | **University of Washington Medical School** | **OHNS-sponsored** | $2,500.00 | Y |  |  |  |  | <https://otolaryngology.uw.edu/education-training/medicalstudents/diversitygrant> |
| Madigan Army Medical Center Program | **None** | **N** |  |  |  |  |  |  |  |
| University of Wisconsin Hospitals and Clinics Program | **University of Wisconsin Medical School** | **OHNS-sponsored** | $4,000.00 |  |  |  | Y |  | <https://www.surgery.wisc.edu/education-training/medical-students/diversity-scholarship/> |
| Medical College of Wisconsin Affiliated Hospitals Program | **Medical College of Wisconsin** | **N** |  |  |  |  |  |  | <https://www.mcw.edu/education/medical-school/prospective-students/visiting-students> |
| West Virginia University Program | **West Virginia School of Medicine** | **N** |  |  |  |  |  |  |  |

Supplemental Table 1. Summary of Identified Visiting Student Funding for Underrepresented in Medicine Students in all Otolaryngology-Head and Neck Surgery Residency Programs in the United States
